# Digital and Dental Malformation and Short Stature in a Patient with Neurological Problems: A Variant of the Oculodentodigital Dysplasia Syndrome or a New Syndrome?

**Published:** 2012

**Authors:** Marjan SHAKIBA, Habibe NEZHAD BIEGLARI, Mohammad Reza ALAEE

**Affiliations:** 1 Assistant Professor of Pediatric Endocrinology, Mofid Children Hospital, Shahid Beheshti University of Medical Sciences, Tehran, Iran; 2 Pediatrician; 3 Associate Professor of Pediatric Endocrinology, Mofid Children Hospital, Shahid Beheshti University of Medical Sciences, Tehran, Iran

**Keywords:** Oculodentodigital dysplasia, White matter lesions, Digital Abnormality

## Abstract

Several syndromes have been recognized with digital abnormality and CNS involvement such as oculodentodigital dysplasia (ODDD), Mohr syndrome and Joubert syndrome. We report a patient who was referred to us because of the neurological signs suspicious of metabolic disorders. This case was a 22-year-old woman whose problems began 4 years ago with shortening of memory, ataxia, abnormal gait and diplopia which progressed slowly.

She consulted many neurologists and was on treatment with the suspicion of vasculitis, but no response was detected. She had severe short stature, hypoplasia of the middle and distal phalanges of the first, second and third fingers, clinodactyly, abnormal toes, abnormal enamel and missing teeth. She had no characteristic faces of ODDD and ophthalmological abnormality. Our patient might be a variant of ODDD or a new syndrome with somatic and neurologic signs.

## Introduction

Several syndromes have been identified with characteristics such as digital abnormality and neurologic involvement. Oculodentodigital dysplasia (ODDD) is an autosomal dominant inherited disorder that affects many parts of the body, particularly the eye (oculo), the teeth (dento) and the fingers (digital). 

In 1920, Lohmann described this syndrome with microphthalmus and camptodactyly of the fifth finger (1), although several descriptions of similar cases with incomplete features were published before 1957 (2-4). Clinical expressions in ODDD patients are highly variable even among members of the same family (5).

Mohr syndrome (orofacial features, polydactyly, halux duplication, brachydactyly, hypotonia and mental retardation) (6) and Joubert syndrome (aplasia of the cerebellar vermis, frontal bossing, hypertelorism, ataxia, syndactyly, abnormal pattern of breathing and jerky eye movement) are other syndromes that involve the face, the digits and CNS development (7). Smith Lemli Opitz is another syndrome with polydactyly, syndactyly, neurological problems and 46, XY disorder of sex development.

We report a patient who was referred to us with prominent neurological signs and abnormal neuroimaging with the suspicion of metabolic disorder. We thought that she might be a variant of ODDD or a new syndrome with short stature, dental and digital malformation and delayed neurological disorders.

## Case Report

The patient was a 22-year-old woman whose problems began 4 years ago with loss of memory, ataxia, abnormal gait and diplopia which progressed slowly. 

She consulted many neurologists and was on treatment with the suspicion of vasculitis, but no response was detected; thus, she was referred to us because of the doubt of inborn error of metabolism. She was born to healthy consanguineous Iranian parents.

She was born with several congenital anomalies, but there was no history of other problems since birth until four years ago when she was affected by ataxia, spasticity, abnormal gait, diplopia and shortening of memory. Some medications were administered in the recent 4 years. Recently, she was on treatment with prednisolone. 

**Figs(1-4).**
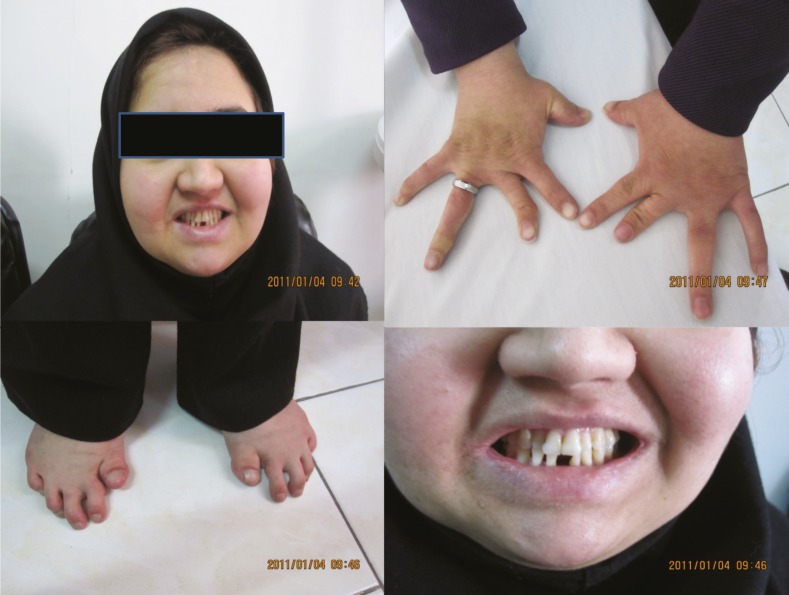
Physical characteristic features of the patient

On examination her height was 142 cm (<3 percentile of growth curve) and her weight was 55 kg. She had microcephaly, prominent round eyes, microdontia, loss of teeth and hypoplasia of the enamel. She had hand, finger and toe anomaly (hypoplasia of the distal and middle phalanges and partial syndactyly of the first, second and third fingers, clinodactyly, wide distance between the fingers, toes and thumbs and hypoplasia. Cubitus valgus was another limb abnormality that was seen on examination (Figures 1-4). She had spastic and ataxic gait (spasticity especially in the lower limbs). Tremor, hyperreflexia and clonus were seen on examination. Cerebellar examination such as the finger to nose and heel to sheen tests was disrupted. She had immature behavior, but normal intelligence.

**Figs(5-8).**
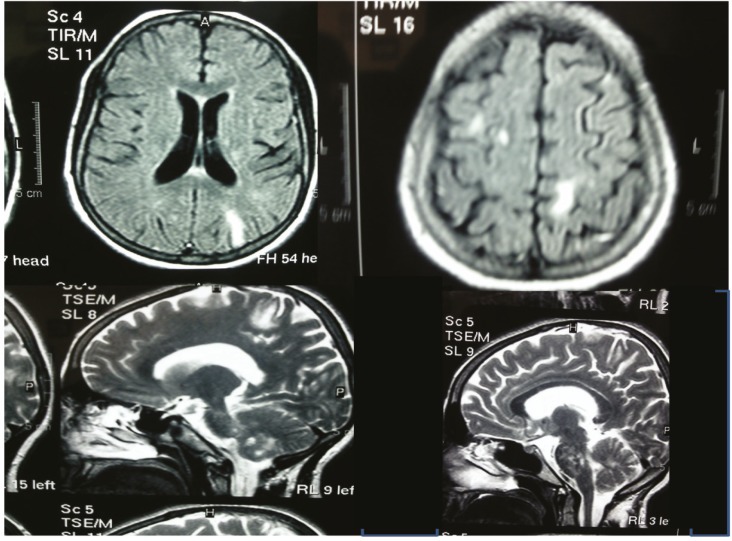
The patient´s neuroimaging

In the brain MRI, hyperintense lesions in the pons, midbrain and white matter were detected. There were demyelinating plaques in the corpus callosum (Figures 5-8). Metabolic causes of white matter disorders such as adrenoleukodystrophy, metachromatic leukodystrophy, globoid cell leukodystrophy had been ruled out for this patient. Routine blood and CSF findings and plasma acylcarnitines and plasma aminoacids were normal. Hexosaminidase A activity was normal.

## Discussion

We report a patient with characteristic features; namely, dental and digital malformation, severe short stature and late onset neurological problems. It seems this patient is a variant form of oculodentodigital dysplasia with incomplete features or a new syndrome with delayed white matter disorders as a characteristic feature. 

Clinical manifestations of ODDD syndrome consist of ophthalmological problems (microphthalmia, microcornea, malformation of the iris, glaucoma and optic atrophy); dental malformation (dental hypoplasia, microdontia, missing teeth, premature loss of teeth and caries); malformation of the hands and feet (type III syndactyly, midpharyngeal and distal pharyngeal hypoplasia, aplasia of the digits or toes, camptodactyly and clinodactyly of the fifth fingers); craniofacial abnormality (short palpebral fissures, hyper or hypotelorism, a narrow nose with hypoplastic alae and a long nasal bridge, thin, anteverted nostrils, wide alveolar ridge and microcephalus); and finally skeletal and hair abnormalities (8). Our patient had hand and feet malformation without type III syndactyly. She had dental malformation similar to ODDD patients, although she did not have the characteristic face and nose of an ODDD patient at all. She did not have microphthalmia and any ophthalmological problems on ophthalmological consult and she had severe short stature, moon face and prominent round eyes as a different feature. ODDD patients have many neurological symptoms maybe delay in onset such as cranial nerve involvement, shortened memory, spasticity, increased reflexes ataxia and atactic gait, muscle weakness dysarthria, tremor, hypotonia dysdiadochokinesia, sensory and autonomic involvement and different reports of retarded or normal intelligence (8). This patient had diplegic spasticity, increased reflexes, ataxia and abnormal cerebellar examinations, diplopia and shortening of memory. The onset of neurological problems was 18 years of age. There was so similarity in neurological signs and symptoms between our patient and ODDD cases. Neuroimaging showed hyperintense lesion in the pons, midbrain and white matter. There were demyelinating plaques in the corpus callosum compatible with some MRI reports of ODDD patients demonstrating white matter changes. Other neuroimaging findings were that basal ganglia and cortex changes were not found in the brain MRI of our patient (8). We only found one ODDD report from Iran in the literature. The patient was a seven-year-old boy with prominent dental problems and facial feature of ODDD (9).

Other differential diagnosis for this case was OFD II (Mohr syndrome) and Joubert syndrome, but we found no similarity between our patient and these disorders except the hand and digit anomaly. Our patient′s neurological findings were also different from these two diseases (6,7). 

We believed our patient is a variant of oculodentodigital dysplasia with an incomplete feature or a new syndrome with dental and digital malformation, short stature and late onset neurological problems.
